# A Novel Ag@AgCl Nanoparticle Synthesized by Arctic Marine Bacterium: Characterization, Activity and Mechanism

**DOI:** 10.3390/ijms232415558

**Published:** 2022-12-08

**Authors:** Shuang Li, Hui Zhang, Bailin Cong, Peiqing He, Wenqi Liu, Shenghao Liu

**Affiliations:** 1 College of Chemistry and Chemical Engineering, Ocean University of China, Qingdao 266100, China; 2 First Institute of Oceanography, Ministry of Natural Resources, Qingdao 266061, China; 3 School of Advanced Manufacturing, Fuzhou University, Fuzhou 350108, China

**Keywords:** Ag@AgCl nanoparticles, synthesis mechanisms, *Shewanella* sp., dye reduction, antibacterial activity

## Abstract

An additive- and pollution-free method for the preparation of biogenic silver and silver chloride nanoparticles (Ag@AgCl NPs) was developed from the bacteria *Shewanella* sp. Arc9-LZ, which was isolated from the deep sea of the Arctic Ocean. The optimal synthesizing conditions were explored, including light, pH, Ag^+^ concentration and time. The nanoparticles were studied by means of ultraviolet-visible (UV-Vis) spectrophotometry, energy dispersive spectrometry (EDS), X-ray diffraction (XRD) and inductively coupled plasma optical emission spectrometers (ICP-OES). The transmission electron microscope (TEM) showed that the nanoparticles were spherical and well dispersed, with particle sizes less than 20.00 nm. With Ag@AgCl nanoparticles, the kinetic rate constants for congo red (CR) and rhodamine B (RhB) dye degradation were 2.74 × 10^−1^ min^−1^ and 7.78 × 10^−1^ min^−1^, respectively. The maximum decolourization efficiencies of CR and RhB were 93.36% and 99.52%, respectively. Ag@AgCl nanoparticles also showed high antibacterial activities against the Gram-positive and Gram-negative bacteria. The Fourier transform infrared spectroscopy (FTIR) spectrum indicated that the O-H, N-H and -COO- groups in the supernatant of Arc9-LZ might participate in the reduction, stabilization and capping of nanoparticles. We mapped the schematic diagram on possible mechanisms for synthesizing Ag@AgCl NPs.

## 1. Introduction

Since they display a high surface-to-volume ratio and exhibit improved properties compared with bulk materials, metal nanoparticles (MNPs) have been extensively applied in catalysis, biological labelling, optoelectronics, surface-enhanced Raman scattering detection, etc [1,2]. Among all MNP-based goods available on the market, silver nanoparticles (AgNPs) show the highest degree of commercialization, accounting for approximately 55.4% [3]. Currently, AgNPs have been widely used for making catalysts for the degradation of environmental pollutants [4], developing new antimicrobial [5], antiviral [6] and anticancer drugs [7], and manufacturing packing materials [8].

Due to the COVID-19 pandemic, it is reasonable to find an agent with long-term sterilization properties and broad-spectrum antimicrobial activity. AgNPs have strong antibacterial activity against a wide range of pathogens and low cytotoxicity towards mammalian cells [9,10]. Notably, the combination of Ag^+^ ions and Cl radicals will further enhance their antibacterial effects [11]. Highly reactive chlorine-free radicals from silver and silver chloride nanoparticles (Ag@AgCl NPs) could efficiently inactivate pathogenic bacteria by attacking the bacterial cell wall and disrupting cellular metabolism [11,12]. However, most studies focus on the antibacterial activities of AgNPs rather than Ag@AgCl NPs.

Conventional physiochemical methods for MNP synthesis involve the use of toxic solvents, harsh chemicals and complicated, expensive techniques, limiting their use in biomedical and clinical fields [13]. Biological nanoparticles are often water soluble and bio-compatible, which are essential properties for many applications [14], especially medical applications, such as coatings for bone prostheses, surgical devices, silver-impregnated catheters, infusion systems and dental composites [3]. To date, AgNPs have been synthesized via biological systems including bacteria, fungi, yeast, viruses, algae and plants [15,16,17]. However, Ag@AgCl NPs are mainly synthesized via chemical methods, such as the ion-exchange process [18], vapour diffusion strategy [19], precipitation method [20] and solvothermal method [21]. The preparation of Ag@AgCl NPs via plant extract is emerging as a new approach, including Saccharum officinarum juice [13], *Aquilaria agallocha* leaf juice [22], *Salvia officinalis* leaf extract [23], *Elaeagnus angustifolia* leaf extract [24], beetroot juice [25] and *Allium sativum* leaf extract [26]. To the best of our knowledge, *Staphylococcus pasteuri* sp. nov., ZAR1, which is isolated from the Zarshouran gold mine, is the only bacteria reported to have the ability to synthesize Ag@AgCl NPs [27]. So, the preparation of Ag@AgCl NPs via bacteria has great significance and broad implications.

As a well-known dissimilatory reducing bacteria, the *Shewanella* genus plays crucial roles in biogeochemical cycles, nanomaterials formation, microbial fuel cells and bioremediation owing to its ability to use a wide range of terminal electron acceptors [28,29,30]. *Shewanella* microbial fuel cells boosted the charge-extraction efficiency substantially by introducing transmembrane and outer-membrane silver nanoparticles [29]. Remarkably, despite the large number of metal nanoparticles synthesized by the *Shewanella* genus, there are very limited reports on Ag@AgCl NPs.

The concrete mechanisms of AgNPs and Ag@AgCl NPs remain unknown. Previous studies suggested that plasmid-mediated silver resistance rendered bacteria capable of accumulating the silver intracellular and silver resistance gene homologues including *silE*, *silP* and *silS* [31,32]. The reductases, reducing agents, amino acids and peptides produced by microorganisms might participate in metal reduction as well as the capping of nanoparticles to a narrow size range [33,34]. However, for extracellular metal ion reduction, microorganisms face the dilemma of how to transfer electrons derived from central metabolisms onto extracellular electron acceptors [35]. The *Shewanella* species appear to be specially adapted for the reduction of extracellular electron acceptors, and the electron transfer mechanisms involve c-type cytochromes, extracellular electron shuttles and direct interspecies electron transfer [35]. Thus, it is significant to investigate the mechanism of nanomaterial synthesis by microorganisms from the perspective of electron transfer.

Here, we synthesized biogenic Ag@AgCl NPs via the cell-free supernatant of *Shewanella* sp. Arc9-LZ in darkness. The bacteria were isolated from the marine sediments of the Arctic Ocean (158°01′12″W; 84°28′38″N). The synthesis conditions of Ag@AgCl were optimized for silver nitrate concentrations, pH and duration. The nanoparticles were studied by means of ultraviolet-visible (UV-Vis) spectrophotometry, transmission electron microscopes (TEM), energy dispersive spectrometry (EDS), X-ray diffraction (XRD) and inductively coupled plasma optical emission spectrometers (ICP-OES). The degradation kinetic rate constants for congo red (CR) and rhodamine B (RhB) dye were calculated, and the antimicrobial activity against Gram-negative (*Pseudomonas aeruginosa* and *Escherichia coli*) and Gram-positive bacteria (*Bacillus subtilis* and *Staphylococcus aureus*) were investigated. Combined with genome annotation, we mapped the electron transfer and synthesis mechanism of Ag@AgCl NPs in *Shewanella* sp. Arc9-LZ.

## 2. Results and Discussion

### 2.1. Identification of the Bacterium

**Figure 1:**
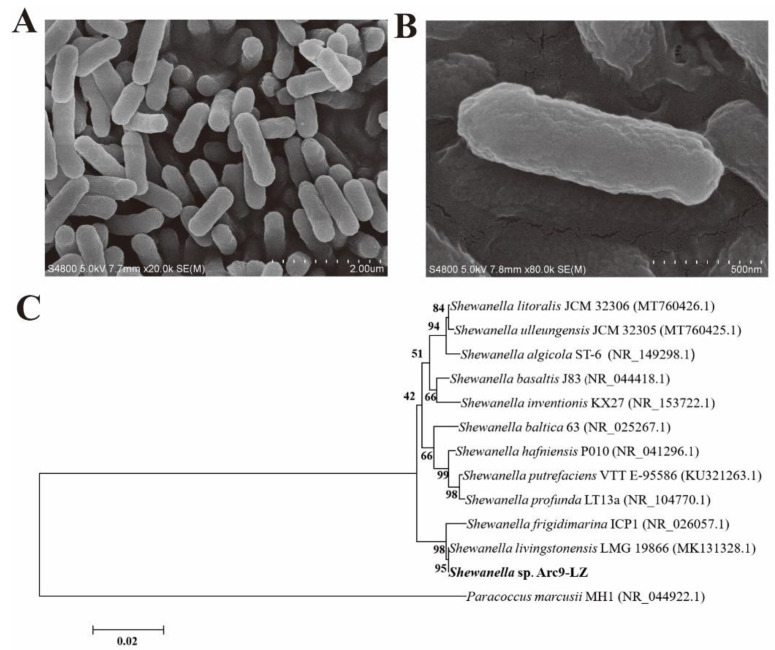
SEM results of the stain *Shewanella* sp. Arc9-LZ (**A**,**B**). Phylogenetic tree based on 16S rRNA gene sequences, Bootstrap ≥ 1000. GenBank accession numbers are indicated in parentheses (**C**).

The scanning electron microscope (SEM) results showed that *Shewanella* sp. Arc9-LZ is rod-shaped, lacks flagella, and is 1.2–1.5 µm in length and 0.25–0.40 µm in width (Figure 1A,B). The 16S rRNA gene sequence of this strain was blasted against the National Centre for Biotechnology Information (NCBI) and showed 99.50% similarity with *Shewanella livingstonensis* LMG 19866 (MK131328.1). The phylogenetic trees based on the 16S rRNA gene sequence also indicated that strain Arc9-LZ was clustered with the genus *Shewanella* and showed the highest similarity with *Shewanella livingstonensis* LMG 19866 (Figure 1C). This strain has been deposited in the China General MicroBiological Culture Collection Centre (CGMCC) with accession number CGMCC 1.18550.

The biological nanoparticles synthesised from the genus *Shewanella* are summarized in Table 1. All the genera mentioned were isolated from warm and tropical regions, including *Shewanella* sp. PV-4 (isolated from deep-sea, Hydrothermal Naha vent, HI, USA) [36], *Shewanella* sp. HN-41 (isolated from tidal flats, Haenam, the Republic of Korea) [37,38], *Shewanella algae bangaramma* (isolated from the coast, Pudhumadam, India) [39], *Shewanella algae* ATCC 51181 (isolated from bottom sediments, Great Bay estuary, New Hampshire) [40,41,42], *Shewanella oneidensis* KR-12 (isolated from Ke-Ya River, Hsinchu, Taiwan) [43] and *Shewanella oneidensis* MR-1 (isolated from Oneida Lake, NY, USA) [28,44,45,46,47,48,49,50]. To the best of our knowledge, no research shows that genus *Shewanella* isolated from cold environments has the ability to reduce metal ions, and Ag@AgCl NPs are a novel material compared to the metal nanoparticle synthesis by *Shewanella* that was previously investigated. It gives clues for understanding the biogeochemical cycles of silver in mid to high ocean latitudes, especially in polar regions.

### 2.2. Synthesis of Biogenic Ag@AgCl Nanoparticles 

**Figure 2:**
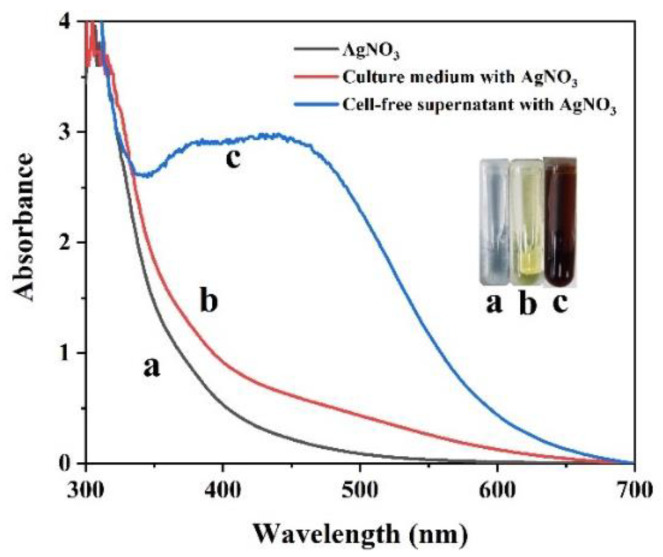
UV/vis spectral analysis of AgNO_3_ (**a**), culture medium with AgNO_3_ (**b**) and the culture supernatant of *Shewanella* sp. Arc9-LZ with AgNO_3_ (**c**) over the wavelength range of 300–700 nm after 48 h of reaction.

Surface plasmon resonance (SPR) excitation in the collective oscillation of free conduction electrons is provoked by an interacting electromagnetic field, leading to colour changes [52]. Thus, AgNP formation could be estimated visually by the observed colour change from light yellow to reddish brown (Figure 2) [53]. The presence of AgNPs rather than AgCl enabled the absorption of light in the visible region, with an absorption peak of approximately 410 nm [13]. This solution presented fine homogeneity, and no precipitation was detected, suggesting that the nanoparticles were stable and well dispersed [54]. No obvious biogenic AgNP formation was detected in the control (only AgNO_3_ and culture medium with AgNO_3_) (Figure 2).

### 2.3. Characterization of Biogenic Ag@AgCl Nanoparticles

**Figure 3:**
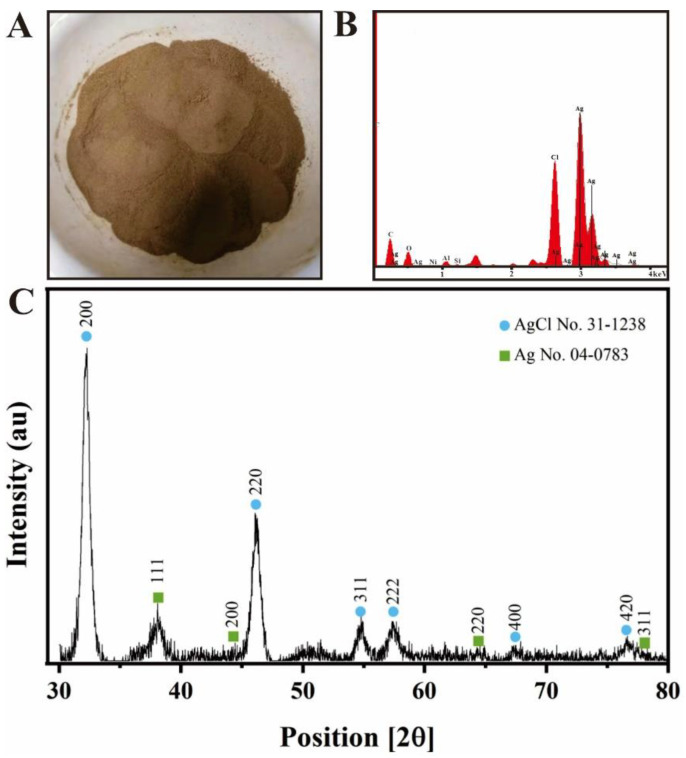
Powder of nanoparticles (**A**), EDS spectrum (**B**) and XRD spectrum (**C**) of biogenic Ag@AgCl nanoparticles.

After freeze-drying, brown-red Ag@AgCl nanoparticle powder was obtained (Figure 3A). On the basis of SPR, optical absorption peaks at 3 keV targeted the presence of metallic silver nanoparticles [55] (Figure 3B). The EDS results illustrated the presence of silver and chlorine elements, accounting for 6.53% and 2.08% of the total Ag@AgCl NPs, respectively (Figure 3B). The strong signals targeting C and O might indicate the presence of proteins acting as capping material on the nanoparticle’s surface [56]. The XRD pattern of the biogenic Ag@AgCl nanoparticles is shown in Figure 3C. It exhibits peaks at 2θ = 32.24, 46.13, 54.88, 57.52, 67.2, 76.8, corresponding well to the (200), (220), (311), (222), (400) and (420) planes of AgCl and matching the JCPDS file 31-1238 for solid AgCl. This spectrum also shows peaks at 2θ = 38.30, 44.01, 64.24, and 77.48, which can be assigned to the (111), (200), (220) and (311) planes, corresponding well to the face-centred cubic structure of metallic silver, matching the JCPDS file 65-2871 for cubic Ag. 

**Figure 4:**
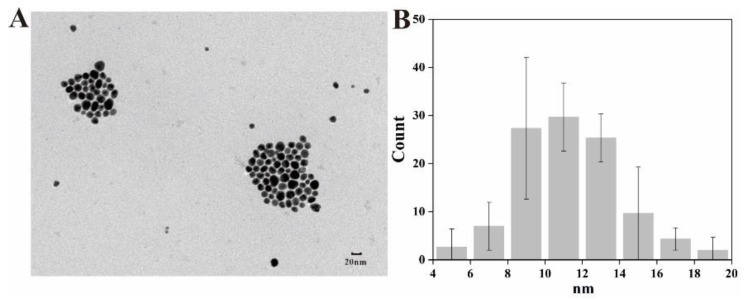
TEM image (**A**) and size distribution (**B**) of Ag@AgCl NPs.

The morphology, size distribution and dispersibility of biogenic nanoparticles are shown in Figure 4. The Ag@AgCl NPs were spherical and ellipsoidal with beneficial dispersibility (Figure 4A). The size ranged from 4 nm to 20 nm, and most nanoparticles concentrated in the size of 8–16 nm, which was in accordance with the normal distribution (Figure 4B). There are limited reports on the biogenic Ag@AgCl NPs, so we summarized the morphology and size of AgNPs synthesized by bacteria (Table 2). Most biogenic nanomaterials from other reports were spherical. The nanoparticles in this study have larger specific surface areas, which may improve the loading of the surfaces or enable a greater release of ions into the solutions [1]. For example, small nanoparticles display larger surface areas than large particles, leading to higher antimicrobial and catalytic activity [57]. The nanoparticles will have great significance when they are uniform in size and shape and are well dispersed [13]. The results demonstrated that *Shewanella* sp. Arc9-LZ cell-free supernatant provided natural capping to synthesize nanoparticles and prevent the aggregation of nanoparticles [34]. 

### 2.4. Influence of Synthesizing Conditions on Ag@AgCl NPs

**Figure 5:**
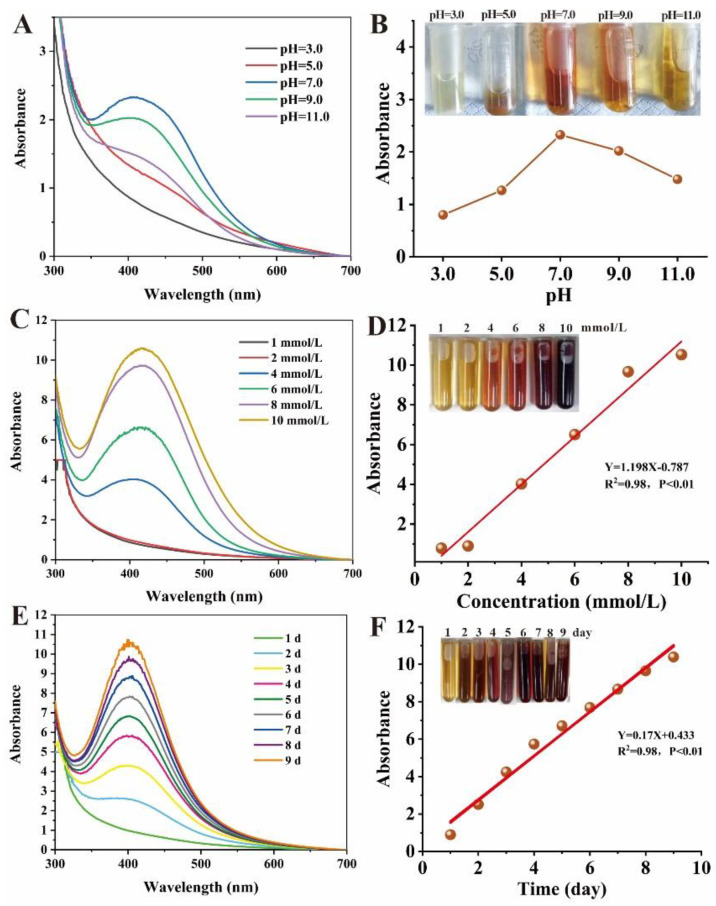
Influence of pH, Ag^+^ concentration and reaction time on nanoparticle synthesis: Uv-vis spectra of reaction system with different pH (**A**), Ag^+^ concentration (**C**) and reaction time (**E**). Digital images and linear graphs of reaction mixtures with different pH (**B**), Ag^+^ concentration (**D**) and reaction time (**F**).

The pH, Ag^+^ concentration and reaction time are important parameters affecting morphology, diameter and dispersity [71,72]. Therefore, we investigated the influence of pH, Ag^+^ concentration and reaction time on biogenic Ag@AgCl NP production (Figure 5). The reaction system has the highest absorbance at 410 nm and the deepest reddish brown under neutral conditions, followed by alkaline and acidic conditions (Figure 5A,B). There was a flocculent precipitate existing in the solution under acidic conditions (pH = 3.0 and pH = 5.0), which may be attributed to the protein denaturation. We concluded that the optimal pH for microbial synthesis of nanoparticles was related to the pH of the microbial environment. As shown in Figure 5C,D, the producing number of AgNP nanoparticles positively correlated with the AgNO_3_ concentration (0–8 mmol/L) added to the cell-free supernatant of Shewanella sp. Arc9-LZ (R^2^ = 0.98, *p* < 0.01). Abundant reducing and capping agents exist in the Arc9-LZ cell-free supernatant. The synthesis efficiency of the Ag nanoparticles increases with increasing Ag^+^ concentration, which may be attributed to the increased probability of the reducing agent colliding with silver ions. As shown in Figure 5E, the SPR intensity at approximately 410 nm increased steadily as a function of reaction time, without an obvious band shift that targets the increase in particle size [73]. Therefore, our results preliminarily indicated that the amount of biogenic Ag@AgCl nanoparticles grew over time (R^2^ = 0.98, *p* < 0.01) and that the size was stable from 1 d to 9 d (Figure 5E,F).

### 2.5. Catalytic Activity of the Biogenic Ag@AgCl Nanoparticles

Dyes and dyestuffs are widely used within the food, pharmaceutical, cosmetic, textile and leather industries [74]. Over 7 × 10^5^ tons of synthetic dyes are produced per year, and approximately 10–15% is discharged into the environment [75]. Both azo and rhodamine B dyes can cause direct destruction of aquatic wildlife and are mutagenic and carcinogenic to humans [76]. Newer and stricter legislation has been established in many countries to enhance enforcement concerning wastewater discharge [75]. Thus, there is a growing need to develop eco-friendly methods to remove dyes from wastewater. Capping agents and stabilizers might lead to the production of small nanoparticles. However, steric hindrance on the surface of the nanoparticle might affect its activities [77]. Hence, it is necessary to verify the catalytic activity of our biogenic Ag@AgCl nanoparticles.

**Figure 6:**
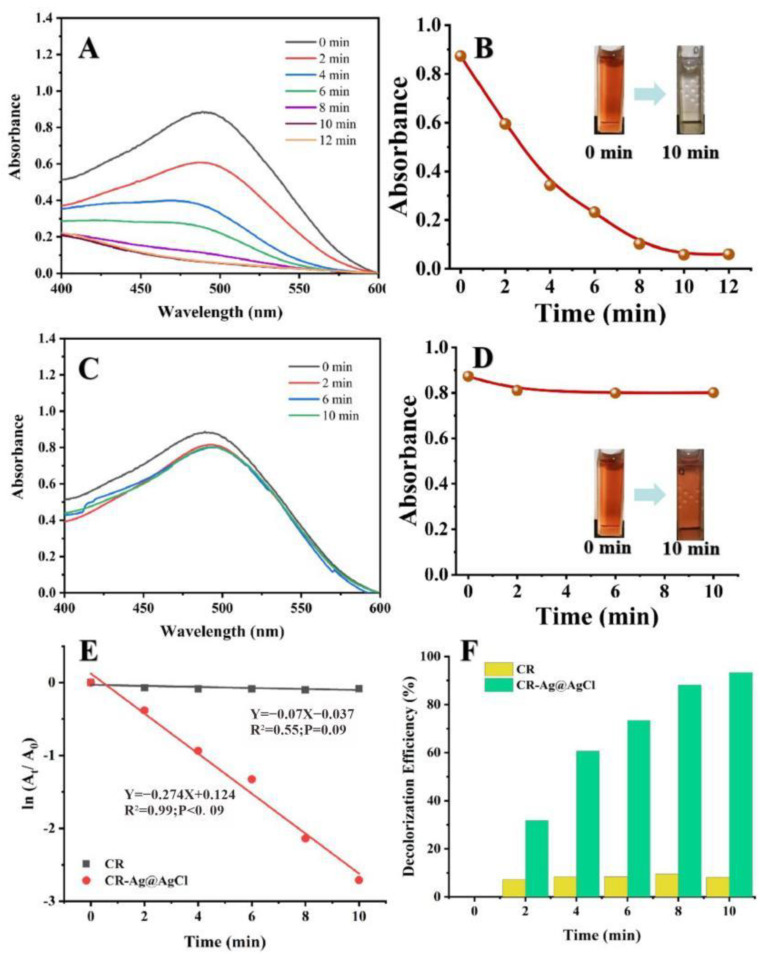
The reduction of CR in aqueous solution recorded every 2 min: the Uv-vis spectral of CR reaction with Ag@AgCl nanoparticles (**A**) and without (**C**); Digital images of CR reaction with Ag@AgCl nanoparticles (**B**) and without (**D**); ln(A_t_/A_0_) versus reaction time for CR reduction (**E**); Decolourization efficiency versus reaction time for CR reduction (**F**).

Figure 6A demonstrates the UV-Vis absorption spectra and colour changes of the CR solution treated with NaBH_4_ in the presence of biogenic Ag@AgCl nanoparticles over a 12 min period. Before degradation, the bright red CR solution showed a strong absorption peak of 497 nm. Upon the addition of 0.025 mg/L (ultimate concentration) biogenic Ag@AgCl nanoparticles into the reaction system, the colour of the solution changed from bright red to colourless within 10 min. Meanwhile, the absorption peak of 497 nm significantly decreased over time. Figure 6C shows the UV-Vis absorption spectra of the CR solution treated with NaBH_4_ in the absence of the Ag@AgCl nanoparticles. It is obvious that there is no change in the colour or the maximum absorption peak of the CR solution. Thus, the degradation of CR by NaBH_4_ is limited in the absence of the Ag@AgCl nanoparticles. The pseudo-first-order linear relation of ln(A_t_/A_0_) versus the reaction time of the degradation of CR is depicted in Figure 6E. According to A_t_ and A_0_, the kinetic rate constant (*k*) of the catalytic reaction in the presence of the biogenic Ag@AgCl nanoparticles is 2.74 × 10^−1^ min^−1^. Figure 6F shows the decolourization efficiency of the CR solution with the Ag@AgCl nanoparticles. The maximum decolourization efficiency of CR by the Ag@AgCl nanoparticles was 93.36% (10 min), which was much higher than the natural decolourization efficiency (9.51%, 8 min) in the absence of Ag@AgCl nanoparticles. We, therefore, suggested that the biogenic Ag@AgCl nanoparticles synthesized by *Shewanella* sp. Arc9-LZ exhibited excellent catalytic activity.

**Figure 7:**
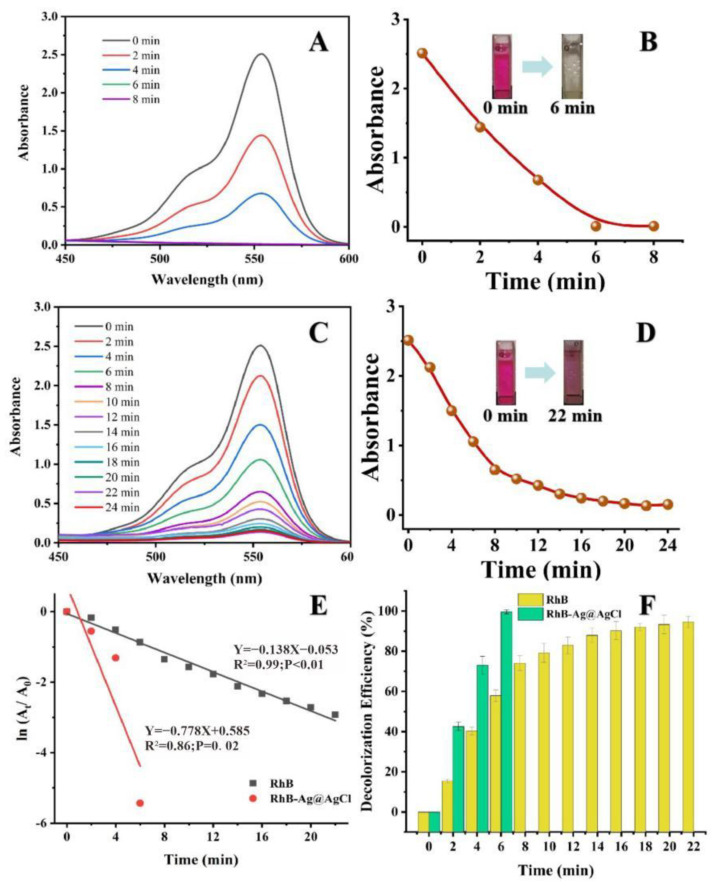
The reduction of RhB in aqueous solution recorded every 2 min: the Uv-vis spectral of RhB reaction with Ag@AgCl nanoparticles (**A**) and without (**C**). Digital images of RhB reaction with Ag@AgCl nanoparticles (**B**) and without (**D**). ln(A_t_/A_0_) versus reaction time for RhB reduction (**E**). Decolourization efficiency versus reaction time for RhB reduction (**F**).

The RhB dye will absorb light at 554 nm in the visible region, showing a bright rose-red colour. After the addition of the biogenic Ag@AgCl nanoparticles to the RhB dye solution, the absorbance intensity at 554 nm showed a sharp decline within 6 min (Figure 7A). In contrast, the absorbance intensity of the dye solution lacking Ag@AgCl nanoparticles remained stable as the reaction time proceeded to 22 min (Figure 7C). Figure 7E indicates that after the addition of NaBH_4_, the kinetic rate constant (*k*) for RhB degradation with Ag@AgCl nanoparticle treatment was 7.78 × 10^−1^ min^−1^, which was higher than that of the control (1.38 × 10^−1^ min^−1^). Additionally, the RhB decolourization efficiency with Ag@AgCl nanoparticle treatment at 6 min was 99.52%, higher than the control of 74.12% (6 min) and 93.91% (22 min) (Figure 7F). It was obvious that the biogenic Ag@AgCl nanoparticles could accelerate the reduction rate of RhB.

The colour fading of the dyes might be attributed to efficient particle-mediated electron transfer from the BH_4_^-^ ion to the dye resulting in the break of azo bonds [78]. With a large surface-to-volume ratio, the nanoparticles expose atoms on the surface as potential catalytic sites [79] and act as a substrate for the electron transfer reaction [80]. They might also indirectly increase the collision probability between the dyes and NaBH_4_ through physical factors [81]. Therefore, the small biogenic Ag@AgCl NPs synthesized by *Shewanella.* sp. Arc9-LZ enables good catalysis.

### 2.6. Antibacterial Activity

**Figure 8:**
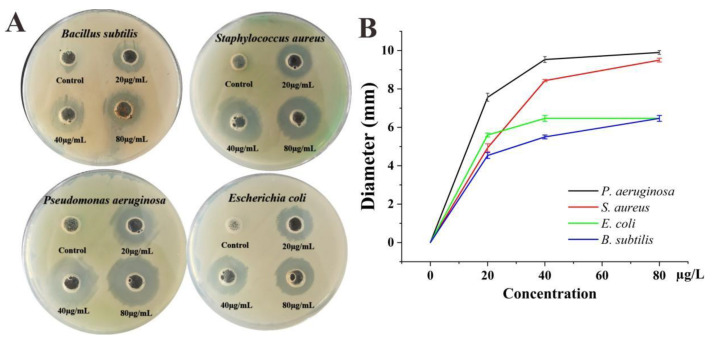
Antimicrobial activities of biogenic Ag@AgCl nanoparticles against *S. aureus*, *P. aeruginosa*, *E. coli* and *B. subtilis*: digital images (**A**) and inhibitory zone diameter (**B**).

The nanoparticles’ antimicrobial ability is not only influenced by the compounds of the material, but it is also related to the material’s size. Smaller nanoparticles have stronger antibacterial effects with larger total surface area per unit volume [82]. In this study, Ag@AgCl nanoparticles showed obvious antimicrobial activity against Gram-positive (Bacillus subtilis ATCC6633 and Staphylococcus aureus ATCC6538) and Gram-negative (Pseudomonas aeruginosa PAO1 and Escherichia coli CGMCC1.2340) bacteria. Compared to the control, the treatments with the Ag@AgCl nanoparticles presented an obvious inhibition zone when the concentrations of nanoparticles reached 20 μg/mL. With increasing Ag@AgCl nanoparticle concentration, the diameter of the inhibition zone tended to increase (Figure 8A,B). Among the strains, Gram-negative Pseudomonas aeruginosa PAO1 had the greatest response to the Ag@AgCl nanoparticles, which can be attributed to the structure of its cell wall.

Ag@AgCl nanoparticles may be related to the release of silver ions, which adhere to the membrane surface, disturb its normal function, cause protein denaturation, affect the respiratory chain and cause irreversible DNA damage, eventually leading to microbial death [9,12]. Tamboli and Lee [83] believed that the changes and damage to the membranes engendered by the Ag@AgCl nanoparticles caused a significant increase in permeability, leaving bacterial cells incapable of properly regulating transport through the plasma membrane and destroying the double-stranded DNA structure, resulting in cell death. Studies have found that the antibacterial effects of silver nanoparticles increased with decreasing particle size [84]. Particles with higher specific surface areas dissolve faster than those with smaller surface areas, which is why smaller particles have higher effective Ag^+^ concentrations and better antibacterial effects [82].

### 2.7. The Mechanism of Nanoparticle Formation

**Figure 9:**
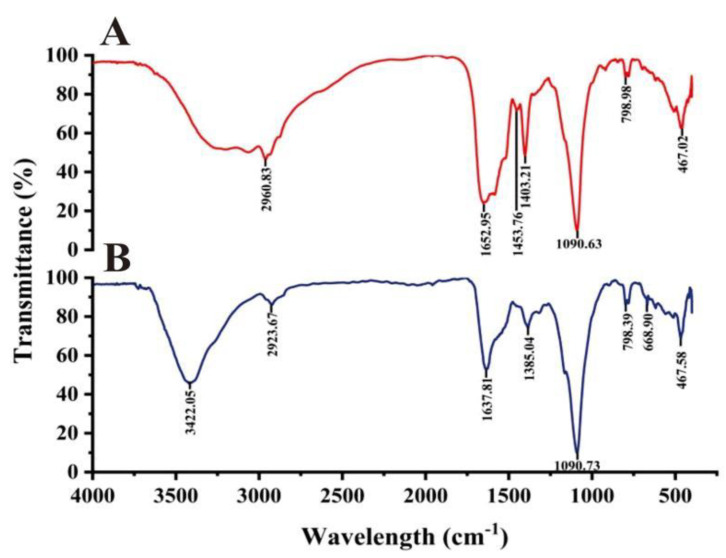
FTIR spectra recorded from microbially synthesized Ag@AgCl nanoparticles: cell-free supernatant (**A**) and biogenic Ag@AgCl nanoparticle solution (**B**).

Duan et al. [33] believed that proteins, amino acids, organic acids and secondary metabolites are related to reducing, capping and stabilizing nanoparticle formation. According to the FTIR spectrum of the cell-free supernatant and biogenic Ag@AgCl solution, the functional groups responsible for the reduction of Ag^+^ were tentatively explored (Figure 9). During the reduction, three bands at 1403.21, 1453.76 and 1652.95 cm^−1^ weakened or disappeared, and two major peaks at 3422.05 and 668.90 cm^−1^ appeared. The stretching vibrations of the O–H and N–H groups are located at 3422.05 cm^−1^ [85]. The bands observed at 1403.21 cm^−1^ and 668.90 cm^−1^ can be assigned to the O–H stretching vibrations of the carboxylate and N–H deformation vibrations of the amine. The band at 1453.76 represents the stretching vibrations of –COO– groups of amino acids with free carboxylate groups [86]. The peak of 1650 cm^−1^ is attributed to carbonyl stretching and is a typical indicator of amide linkages [87]. The FTIR spectra showed that amino acids, proteins and organic molecules with amide linkages in the supernatant of Arc9-LZ might participate in the reduction, stabilization and capping of the biogenic Ag@AgCl nanoparticles.

**Figure 10:**
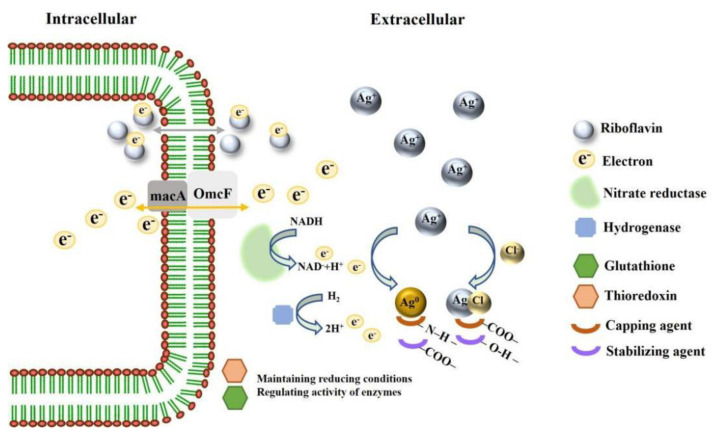
Schematic diagram on mechanisms of synthesizing Ag@AgCl NPs.

To speculate the mechanism of Ag@AgCl NPs synthesis, we summarized the genes, enzymes, proteins and small molecules that may be involved in the synthesis of Ag@AgCl NPs in Table 3. The complete genome sequence of *Shewanella* sp. Arc9-LZ has been submitted to the GenBank database under accession number CP048031 [88]. The whole genome of *Shewanella* sp. Arc9-LZ was annotated by databases of Kyoto Encyclopedia of Genes and Genomes (KEGG), Cluster of Orthologous Groups of proteins (COG), Non-Redundant Protein Database (NR), Transporter Classification Database (TCDB), Swiss-Protand Database and Carbohydrate-Active enZYmes Database (CAZy) [88]. We concluded that the mechanism of Ag@AgCl NP synthesizing by *Shewanella* sp. Arc9-LZ is not plasmid-mediated silver resistance but extracellular electron shuttles. The schematic diagram of mechanisms is shown in Figure 10, which needs further evidence.

## 3. Materials and Methods

### 3.1. Materials

The strains *Bacillus subtilis* ATCC6633, *Staphylococcus aureus* ATCC6538, *Pseudomonas aeruginosa* PAO1 and *Escherichia coli* CGMCC1.2340 were used for assaying antibacterial activities and were stored at −80 °C. The dyes CR and RhB were purchased from Sangon Biotech (Shanghai, China) and Aladdin (Shanghai, China), respectively. NaBH_4_ was supplied by Sinopharm Chemical Reagent Co., Ltd. (Shanghai, China). AgNO3 was obtained from the Shanghai Chemical Reagent Factory (Shanghai, China). The yeast extract, tryptone and agar powder were all purchased from Solarbio Ltd. (Beijing, China).

### 3.2. Isolation and Identification of the Strain Shewanella *sp*. Arc9-LZ

*Shewanella* sp. Arc9–LZ bacteria were isolated from the marine sediments of the Arctic Ocean (158°01′12’’W; 84°28’38’’N) collected during the 9th Chinese National Arctic Expedition in 2018. Firstly, *Shewanella* sp. Arc9-LZ was activated in the marine ZoBell 2216E medium (peptone, 5 g/L; yeast extract, 1 g/L; natural seawater, 1 L) at 150 r/min. After incubation at 15 °C for 2 d, the culture was serially diluted and spread on 2216E medium agar plates (ZoBell 2216E medium with 15 g/L agar powder) to isolate the single clone.

Based on the 16S rRNA sequence alignment, the strain was identified by PCR with the Bact27F and Univ1492R primers. PCR amplicons of 16S rRNA genes were sequenced by Sangon Biotech (Shanghai, China). Sequences (1500 bp fragments) were analysed using CodonCode Aligner software. Additionally, 16S rRNA gene sequences were aligned with the closest matches available in GenBank and EzTaxon server 2.1 with the Clustal W function of BioEdit software (7.1.3.0). Phylogenetic trees were constructed with the Molecular Evolutionary Genetics Analysis software (MEGA version 4.0) using the neighbour-joining method, and 1000 bootstraps were performed to assign confidence levels to the tree nodes.

The DNA of this stain was extracted with a Bacteria DNA kit (TIANamp, DP302, Tiangen Biotech, Beijing, China). Gene Pools were constructed on the Pacbio platform with an SMRT bell TM Template kit (version 1.0) and Illumina PE150 platform with NEBNext^®^Ultra™ DNA Library Prep Kit for Illumina (NEB, Ipswich, Ipswich, MA, USA), respectively. The sequence was analysed by PacBio Sequeland Illumina NovaSeq PE150 for different libraries [88]. 

### 3.3. Biosynthesis of the Silver Nanoparticles in the Dark

*Shewanella* sp. Arc9–LZ was cultured in the YP medium (peptone, 10 g/L; yeast extract, 5 g/L; ultra-pure water, 1 L) and incubated at 150 r/min and 15 °C for 48 h. To obtain 100 mL of cell-free extracts, the fermented liquid was centrifuged at 12,000 r/min for 15 min before being filtered through a 0.22 µm syringe filter. Then, biogenic Ag@AgCl nanoparticles were synthesized by mixing the cell-free supernatant and AgNO_3_ at 150 r/min and 35 °C in the dark. The cell-free supernatant without AgNO_3_ and the liquid media with AgNO_3_ were kept under the same conditions and set as controls.

### 3.4. Characterization of Biogenic Ag@AgCl Nanoparticles

To characterize biogenic Ag@AgCl nanoparticles, samples were monitored using ultraviolet-visible (UV-Vis) spectrophotometry (Shimadzu Model UV 2550, China) in the range of 300–700 nm at a resolution of 1 nm. The biogenic Ag@AgCl nanoparticles were designed under different pH conditions (pH = 3.0, 5.0, 7.0, 9.0, 11.0), final concentrations of AgNO_3_ (1 mmol/L, 2 mmol/L, 4 mmol/L, 8 mmol/L and 10 mmol/L) and lengths of time (1 d, 2 d, 3 d, 4 d, 5 d, 6 d, 7 d, 8 d and 9 d). The morphology and size of the produced Ag@AgCl nanoparticles were discerned by means of transmission electron microscopy (TEM) (Hitachi HT7700, Tokyo, Japan). The presence and structure of the biogenic Ag@AgCl nanoparticles in the samples were determined by means of X-ray diffraction (XRD) (Bruker D8 Advance, Karlsruhe, Germany) and energy dispersive spectrometry (EDS) (IXRF, USA). Fourier transform infrared spectroscopy (FTIR) spectra were obtained on a Nicolet iN10 (Thermo Electron Scientific Instruments LLC, Madison, WI, USA) with wavelengths ranging from 500 to 4000 cm^−1^. The concentration of biogenic Ag@AgCl nanoparticles was analysed by means of inductively coupled plasma optical emission spectrometry (ICP-OES) (Perkin-Elmer Optima, Waltham, MA, USA).

### 3.5. Catalytic Activity for Azo Dyes and RhB

The catalytic activity of biogenic Ag@AgCl nanoparticles for dye reduction was carried out as follows. Firstly, 10 mL of 100 mg/L dyes (CR and RhB), 10 mL of 50 mmol/L NaBH_4_ and 30 mL of ultrapure water were mixed in a conical flask at 40 °C in the dark. Two reaction setups were employed at the same time. In the first setup, biogenic Ag@AgCl nanoparticles were present in the reaction system at a final concentration of 0.025 mg/L. In the second setup, an equal volume of ultrapure water instead of biogenic Ag@AgCl nanoparticles was put into the reaction system as a control. At pre-determined time intervals, a UV-Vis spectrophotometer recorded the reduction of CR and RhB in the ranges of 400–600 nm and 450–600 nm, respectively. Considering that the amount of added NaBH_4_ was much larger than that of the dyes, the catalytic reduction should follow pseudo-first-order kinetics, which can be expressed as ln(A_t_/A_0_) = −kt [72]. In this equation, At represents the dye absorbance at time t, A_0_ represents the initial dye absorbance and slope k represents the apparent reduction rate constant [52].

### 3.6. Antibacterial Activity

The antibacterial activity of biogenic Ag@AgCl nanoparticles was based on the standard agar-well diffusion method with some modifications [95]. The tested strains were cultured in the LB medium (peptone, 10 g/L; yeast extract, 5 g/L; NaCl, 10 g/L, ultra-pure water, 1 L) and spread uniformly on LB agar plates with cotton swabs at a final concentration of 1 × 10^5^–1 × 10^6^ CFU/mL. A sterile cork borer was used to punch 4 circular holes of 8 mm diameter into the plates, and 100 µL of various concentrations of Ag@AgCl (0, 20, 40, 80 µg/mL) was added to the holes. The negative control in the antibacterial study was the LB medium with the same volume. The plates were incubated at 37 °C for 12 h for the observation and calculation of the inhibition zone.

## 4. Conclusions

Ag@AgCl NPs, which are a novel material, were synthesized by the strain *Shewanella* sp. Arc9-LZ, which was isolated from the Arctic Ocean. The methods are eco-friendly (no additional chemical reductant, low energy consumption and waste emission), simple (mild reaction conditions, one-pot process), repeatable, sustainable and renewable. With a small size (≤20 nm), spherical shape and beneficial dispersity, Ag@AgCl nanoparticles exhibited excellent catalysis and antibacterial ability application prospects. The kinetic rate constants (k) for CR and RhB degradation with Ag@AgCl nanoparticles were 2.74 × 10^−1^ min^−1^ and 7.78 × 10^−1^ min^−1^, and the maximum decolourization efficiency of CR and RhB were 93.36% and 99.52%, respectively. Moreover, Ag@AgCl nanoparticles showed high antibacterial ability against the Gram-positive and Gram-negative bacteria investigated. In addition to excellent material and good applications, this method might give clues for the further development of the synthesis of new biomaterials, utilization of abundant microbial resources from the Arctic, explanation of the origin of deep-sea metal resources, and understanding of life and the biogeochemical silver cycle in aqueous environments. The schematic diagram of mechanisms for the synthesis of Ag@AgCl NPs was mapped.

## Figures and Tables

**Table 1:** The summary of biological nanoparticle synthesizing from the genus *Shewanella*.

Bacteria	Size (nm)	Shape	Type of Nanoparticle	Location	Reference
*S*. sp. HN-41	140–221	Spherical	Se	Intracellular	[37]
	10–30	Short rod	Magnetite, siderite	Intracellular	[38]
*S*. sp. PV-4	-	-	Magnetite	Extracellular	[36]
	>35	-	magnetite	Intracellular	[51]
*S. algae* bangaramma	5–30	Spherical	Ag	Intracellular	[39]
*S. oneidensis* KR-12	2–8	Spherical	Pb	Intracellular	[43]
*S. algae* ATCC 51181	5	-	Pt	Intracellular	[41]
	10–20	Spherical, triangle	Au	Extracellular	[40]
	40–50	Rectangular, rhombic, hexagonal	Fe_3_O_4_	Extracellular	[44]
	2–50	Spherical	Au	Extracellular	[45]
	-	Spherical	Pd	Intracellular	[46]
	nano-sized	Needle, hexagonal	Jarosite	Extracellular	[47]
	30–43	Pseudohexagonal, irregular, rhombohedral	Fe_2_O_3_	Intracellular	[28]
	17–23	Spherical	Ag and Au	Extracellular	[48]
			V	Intracellular	[49]
	nano-sized	Amorphous round	Manganese oxide	Intracellular	[30,50]

**Table 2:** The summary of AgNP synthesis from the bacteria.

Stains	Location	Morphology	Size (nm)	Reference
*Acinetobacter calcoaceticus* PUCM 1005	Extracellular	Spherical	4–40 nm	[58]
*Pseudomonas stutzeri* AG259	Intracellular	Subglobose	5–25 nm	[59]
*Bacillus subtilis* MSBN 17	Intracellular	Spherical	60 nm	[60]
*Ureibacillus thermosphaericus*	Extracellular	Spherical	10–100 nm	[61]
*Pseudomonas aeruginosa* ATCC 27853	Extracellular	Spherical	33–300 nm	[62]
*Streptacidiphilus durhamensis* HGG16n	Extracellular	Spherical	8–48 nm	[63]
*Paracoccus* sp. Arc7-R13	Extracellular	Subglobose	2–25 nm	[64]
*Staphylococcus epidermidis* ATCC 12228	Intracellular	Spherical, oval, short rod, triangle	10–100 nm	[65]
*Klebsiella pneumoniae*	Extracellular	Spherical	5–32 nm	[66]
*Aeromonas* sp. THG-FG1.2	Extracellular	Spherical	8–16 nm	[67]
*Actinomycetes* MRS- 1	Extracellular	Spherical	4.7–18.8 nm	[68]
*Fusarium oxysporum*	Extracellular	Spherical	40 nm	[69]
*Trichoderma* spp.	Intracellular	Spherical	14–25 nm	[70]

**Table 3:** Microbial substances that may be involved in the synthesis of silver nanomaterials.

Substances	Functions	Reference
Silver-binding gene homologue: *silE*	Encoding a periplasmic silver-binding protein that presents histidine sites for silver ion binding.	[32]
Three major gene homologues of *silE*, *silP*, and *silS*	Participation in silver resistance	[31]
Periplasmic c-type cytochrome (MacA) and outer membrane c-type cytochrome (OmcF)	Reduction of Ag^+^ to Ag^0^	[89]
NADH-dependent enzymes, especially nitrate reductase	Roles in AgNP synthesis	[90]
Reducing enzymes belonging to class of nitrogenase and hydrogenase	Reduce silver ions to nano-silver	[91]
Cellular nitrogenase	Enzyme concentration dictating the size of AgNPs	[91]
NfsA, an oxygen-insensitive nitroreductase	Reducing AgNO_3_ to AgNPs	[66]
Spore-associated enzymes, like glucose oxidase, alkaline phosphatase, laccase and catalase	Generating the reducing cofactors and stimulating the biogenesis of AgNPs	[92]
Glutathione and thioredoxin systems	Maintaining the reducing conditions indirectly and regulating the activity of enzymes	[93]
Riboflavin	Acting as a soluble redox shuttle to mediate metal reduction	[94]

## Data Availability

The *Shewanella* sp. Arc9-LZ has been deposited in the China General MicroBiological Culture Collection Centre (CGMCC) with accession number CGMCC 1.18550. The complete genome sequence of this strain has been submitted to the GenBank database under accession number CP048031.
